# Transferrin-targeting redox hyperbranched poly(amido amine)-functionalized graphene oxide for sensitized chemotherapy combined with gene therapy to nasopharyngeal carcinoma

**DOI:** 10.1080/10717544.2019.1642421

**Published:** 2019-07-25

**Authors:** Tao Liu, Jingzhen Li, Xidong Wu, Siyi Zhang, Zhongming Lu, Guanxue Li, Junzheng Li, Shaohua Chen

**Affiliations:** a Department of Otolaryngology-Head and Neck Surgery, Guangdong Provincial People's Hospital, Guangdong Academy of Medical Sciences, Guangzhou, China;; b Department of Nephrology, Ningbo Yinzhou Second Hospital, Ningbo, China;; c Department of Pharmacology, Jiangxi Testing Center of Medical Instruments, Nanchang, China;; d Department of Pediatric Center, Zhujiang Hospital of Southern Medical University, Guangzhou, China;; e Department of Otolaryngology-Head and Neck Surgery, The Affiliated Dongguan Hospital of Jinan University, Dongguan, China

**Keywords:** GSH-response, transferrin, graphene oxide, drug and gene co-delivery, sensibilization, nasopharyngeal carcinoma

## Abstract

A drug and gene co-delivery system with chemotherapeutic sensibilization was prepared and used for nasopharyngeal carcinoma therapy. For this purpose, the graphene oxide (GO) was conjugated with the redox hyperbranched poly(amido amine) (HPAA) and then the targeting molecule, transferrin (Tf), was also conjugated. The obtained Tf-HPAA-GO could co-deliver docetaxel (DOC) and MMP-9 shRNA plasmid (pMMP-9) effectively and showed the targeting effect to HNE-1 cells. The co-delivery system showed the effective drug and gene delivery ability with high cytotoxicity and gene transfection efficiency. Besides that, Tf-HPAA-GO/DOC also showed the chemotherapeutic sensibilization effect, the formulation containing HPAA segments showed much higher cytotoxicity than free DOC. Benefiting from the sensibilization effect and DOC/pMMP-9 co-delivery strategy, this Tf-HPAA-GO/DOC/pMMP-9 co-delivery system exhibited the significantly improved therapeutic efficacy to HNE-1 tumor in a combined manner which was confirmed by *in vitro* and *in vivo* assays. This strategy provided an easily delivery system combining the drug/gene co-delivery, chemotherapeutic sensibilization, and targeting into one single platform, which showed a promising application in cancer therapy.

## 1. Introduction

Nasopharyngeal carcinoma (NPC) is a high incidence of tumors in Southern China (Huang et al., 2018). Although chemotherapy is a prevailing and effective strategy for NPC treatment in clinical, it is restricted by side effects, such as the serious adverse effects, drug resistance, and others (Ruuskanen et al., 2018). As solutions, the drug delivery systems which could alter the pharmacokinetics and biodistribution of drugs as well as reduce the side effects of drugs have developed (Zheng et al., 2018; Ruskowitz and DeForest, 2018; Yu et al., 2018). However, there are still two concerns to be resolved. One is that the drug loading capacity of the normal drug delivery systems is insufficient, and the other is that the therapeutic effect needs more improvements (Chater et al., 2018).

For the first concern, graphene oxide (GO) has been explored as the drug carrier in recent years due to its high capacity to the benzene-containing drugs through the π-π interactions (Su et al., 2018). Moreover, the good biocompatibility *in vitro* and *in vivo* of GO has been confirmed by recent reports, gradually dispelling the doubts about its biosafety by the worldwide researchers (Chang et al., 2011; Zhang et al., 2011; Seabra et al., 2014; Wang et al., 2018).

For the second concern, to improve the therapeutic effects to cancers, two strategies have been usually taken into consideration: one is that combining a sensitizer with chemotherapy drugs to enhance the sensitivity of tumor cells to drugs as well as overcome their multidrug resistance, and the other is that combining with gene therapy for a synergistic or adjuvant therapy.

Glutathione (GSH) has been found to be much higher were expected level expression in tumor cells than that in normal cells, and the GSH-mediated detoxification is an important reason for the drug resistance of cancer cells (Jing et al., 2018). Therefore, many GSH-response drug delivery systems have been designed, which were expected to be degraded intracellularly due to the presence of GSH at a high concentration and then achieve the effective drug delivery and rapid drug release (Cheng et al., 2018; Ji et al., 2018; Zhu et al., 2018). Moreover, the drug sensitizers, such as buthionine sulfoximine (BSO), selectively inhibit the synthesis of GSH, have been combined with chemotherapeutic drugs and used in clinical for effective cancer therapy (Hu et al., 2018; Moghaddam et al., 2018; Zhang et al., 2018). Tang et al. have reported a drug carrier combining chemotherapeutic drug sensitization with GSH-response recently (Tang et al., 2018). They synthesized the redox hyperbranched poly(amido amine) containing disulfide bonds (HPAA) and then conjugated the antitumor drug of methotrexate. The obtained prodrug showed the self-sensibilization effect coming from the intracellular GSH consumption resulted from the HPAA segments, and showed effective tumor cell inhibition *in vitro* and *in vivo*.

Gene therapy, mediating the relevant proteins expressions and then affecting the tumor cells, has been a promising strategy for cancer treatment in the past decades. This strategy could avoid the drug resistance and adverse the side effects, which could be combined with chemotherapy (Zhou et al., 2018). Nowadays, many co-delivery systems for drug and gene have been developed (Zhou et al., 2016; Li et al., 2018; Lv et al., 2018).

In this work, a strategy combining the sensitized chemotherapy with gene therapy was developed. The GSH-response cationic HPAA was used to modify GO to obtain HPAA-GO. The docetaxel (DOC), a clinical drug for NPC therapy, was loaded with a high loading amount through the π-π interactions between GO and DOC. And the therapeutic gene, MMP-9 shRNA plasmid (pMMP-9), closely related to tumor cells apoptosis and metastasis (Endo et al., 2018), was encapsulated through the electrostatic interaction between pMMP-9 and HPAA segments. To further improve the therapeutic effect, a targeting molecule, transferrin (Tf), which has been reported to target to NPC cells (Liu et al., 2018), was then conjugated with HPAA-GO. In this work, we developed an “all-in-one” drug delivery system comprised of sensitized chemotherapy, gene therapy as well as intracellularly GSH-triggered degradation and rapid drug/gene release.

## 2. Experimental section

### 2.1. Materials

Cystamine dihydrochloride, 1-(2-aminoethyl) piperazine, acryloyl chloride, and docetaxel were purchased from Aladdin Industrial Corporation (Shanghai, China). Graphene oxide powder was obtained from Aladdin Industrial Corporation and was dried before use. *N*-(3-dimethylaminopropyl)-*N*′-ethylcarbodiimide hydrochloride (EDC•HCl) and *N*-hydroxysuccinimide (NHS) were purchased from Sigma and used directly. The branched polyethylenimines (bPEI, molecular weight of 25 kDa) were purchased from Sigma-Aldrich. All other reagents and solvents were purchased from Aladdin Industrial Corporation and used directly. A pcDNA3 plasmid was designed to construct the vector that expressed the small interference RNA for MMP-9 protein with the enhanced green fluorescent protein (EGFP). This plasmid was obtained from LongSee-Med Technology Co., Ltd. (Guangzhou). Cell counting kit-8 (CCK-8) was obtained from Beyotime Institute of Biotechnology (Shanghai, China). Dulbecco’s Modified Eagle’s Medium (DMEM), trypsine-EDTA (0.25%), and fetal bovine serum (FBS) were obtained from Gibco (BRL, MD, USA). The human nasopharyngeal carcinoma HNE-1 cells were supplied by Southern Medical University (China). Male specific pathogen-free BALB/c nude mice (age of 4 weeks, weight of 18–20 g) were obtained from the Center for Laboratory Animal Sciences, Southern Medical University (license number: SCXK (Yue) 2016-0041) and fed in the Experimental Animal Center of Jiangxi Testing Center of Medical Instruments (Jiangxi Institute of Materia Medica). The Institutional Administration Panel for Laboratory Animal Care approved the experimental design.

### 2.2. Synthesis of HPAA

HPAA was synthesized according to the reported methods (Li et al., 2016). The chemical structure of HPAA was characterized by ^1^H NMR using D_2_O as the solvent. And the gel permeation chromatography (GPC) was performed to analysis the molecular of HPAA using the poly(ethylene oxide) as the standard and 0.8 mol/L aqueous NaNO_3_ solution as the elution liquid with a flow rate of 1 mL/min.

### 2.3. Preparation of HPAA-GO

For HPAA-GO preparation, 100 mg of GO was firstly mixed with 70 mg of EDC•HCl and 40 mg of NHS in 10 mL of PBS (pH = 6.0) at room temperature for 2 h. Then, another 10 mL of PBS solution containing 500 mg of HPAA was added dropwise to the activated GO with stirring followed by a 72-hour reaction. After that, the resultant mixture was dialyzed for 3 days using dialysis membrane (MWCO = 30,000) against the deionized water and then lyophilized to obtain the HPAA-GO with a yield of 68%.

The chemical structure of HPAA-GO was characterized by Raman spectrum. The composition of HPAA-GO was analyzed using thermal gravimetric analyzer with a temperature range of 50–700 °C and a heating rate of 10 °C/min.

### 2.4. Preparation of Tf-functionalized HPAA-GO (Tf-HPAA-GO)

For Tf-functionalization, HPAA-GO was activated first by sulfo-SMCC by mixing 300 mg HPAA-GO PBS solution with 10 mg sulfo-SMCC PBS solution. After 24 h of reaction, the product was dialyzed for 12 h using dialysis membrane (MWCO = 8000) in the distilled water to remove the unreacted sulfo-SMCC. Then, the pH value of the mixture was adjusted to 6.5, and 30 mg Tf-SH was added. The mixture was then reacted at room temperature for another 48 h. After the reaction, the pH value of the mixture was adjusted to 5.9–6.0, followed by centrifugation at 20,000 rpm for 10 min and lyophilization to obtain Tf-HPAA-GO. The Tf content in Tf-HPAA-GO was determined by the Micro BCA Protein Assay Kit with the standard (*R* ^2^ > 0.98).

### 2.5. GSH response

For studying the GSH-response ability of Tf-HPAA-GO, 10 mg Tf-HPAA-GO was dispersed in 10 mL distilled water and then 1 mL aqueous GSH (100 mmol/L) was added. The mixture was stirred at 37 °C for 24 h. The particle sizes and zeta potentials of GO, Tf-HPAA-GO, and Tf-HPAA-GO in the presence of 10 mmol/L GSH were recorded by DLS (Zetasizer Nano-ZS, Malvern, UK) with a monochromatic coherent He-Ne laser. All values were derived from CONTIN program.

### 2.6. DOC delivery

#### 2.6.1. DOC loading

For DOC loading, 25 mg DOC was firstly dissolved in DMSO with the concentration of 5 mg/mL. Then, 100 mg Tf-HPAA-GO was dispersed in 10 mL distilled water in an ice-bath condition. After that, the DOC solution was mixed with Tf-HPAA-GO dropwise. The resultant mixture was stirred at 37 °C for 12 h, and then dialyzed using dialysis membrane (MWCO = 500) against the deionized water for 2 days and lyophilized to obtain the DOC-loaded Tf-HPAA-GO (Tf-HPAA-GO/DOC). The loading amount of DOC in Tf-HPAA-GO/DOC was analyzed using HPLC analysis (Ma et al., 2014).

#### 2.6.2. *In vitro* DOC release

For the *in vitro* DOC release assays, 10 mg Tf-HPAA-GO/DOC enclosed in the dialysis bag (MWCO = 2000) was immersed in 5 mL of PBS (pH = 7.4) containing 10% Tween 80 at 37 °C (Liu et al., 2016). The sample immersed in PBS containing 10 mmol/L GSH was set as the control group. All of the release studies were carried out in triplicate. At the predetermined time points, 2 mL of the release media was taken away for measurements and another fresh 5 mL media was added back to maintain the original medium volume. The amount of the released DOC was determined by HPLC analysis.

#### 2.6.3. Cytotoxicity

The cytotoxicity of Tf-HPAA-GO/DOC was evaluated by CCK-8 assay using HNE-1 cells. The HNE-1 cells were cultured onto a 96-well plate (5 × 10^3^ cells per well) in a complete DMEM (with 10% fetal bovine serum supplemented and high glucose) at 37 °C in a humidified atmosphere with 5% CO_2_. After overnight incubation, 100 µL complete DMEM that contained the desired amount of Tf-HPAA-GO/DOC replaced the growth medium, and each sample was set for five multiple holes. The same amount of PBS was used as the control group. After 24 h incubation, 100 µL fresh DMEM with 10 µL of CCK-8 per wells were incubated in the cells for another 2 h. The absorbance at a wavelength of 450 nm in each well was measured by a microplate reader to calculate the number of viable cells. For HPAA sensitized DOC therapy assay, free DOC was also used the as control group compared with Tf-HPAA-GO/DOC and HPAA-GO/DOC.

Moreover, in order to explore the targetability of Tf, Tf-HPAA-GO/DOC and HPAA-GO/DOC were incubated with HNE-1 cells for only 4 h and then cultured with DMEM for a further 24 h. The cytotoxicity was determined by CCK-8 assay.

### 2.7. pMMP-9 delivery

#### 2.7.1. Tf-HPAA-GO/pMMP-9 complexes

Tf-HPAA-GO and pMMP-9 were co-dissolved in pure water to make the suitable concentrations of aqueous solution. The resulting aqueous components were mixed at 25 °C, and gently stirred for 10 min to form the Tf-HPAA-GO/pMMP-9 complexes.

#### 2.7.2. Gel electrophoresis

The binding ability of Tf-HPAA-GO to pMMP-9 was evaluated by gel electrophoresis assay. TAE buffer (1 mmol/L EDTA and 40 mmol/L Trisacetate) was used to prepare the agarose gel (1.0%, w/v) containing the ethidium bromide (0.25 mg/mL). After an incubation for 15 min at 25 °C, all samples were performed by electrophoresis on the agarose gel at 70 V for 20 min. Visualization and image capture were accomplished using the UV-trans illuminator under a Kodak EDAS 290 digital imaging suite (Fisher Scientific, PA).

#### 2.7.3. *In vitro* transfection

HNE-1 cells were seeded into the 24-well culture plates using 500 µL complete DMEM as the culture medium at a density of 4 × 10^4^ cells per well. After 12 h incubation, the culture medium was interchanged by fresh Tf-HPAA-GO/pMMP-9 complexes (weight ratios of from 10 to 30) in Opti-DMEM. The pMMP-9 amount in each well was fixed at 2.0 µg. The cells were incubated for another 36 h and then analyzed by green fluorescent protein (GFP) expression using a fluorescence microscope (Nikon-2000U, Japan). The cells treated with PEI-25k/pMMP-9 (weight ratio of 1.3) and HPAA-GO/pMMP-9 complexes were set as the control groups. After the cells were digested by trypsinase, the transfection percentages (positive cell percent) were recorded by a flow cytometer (BD Accuri C6).

#### 2.7.4. MMP-9 protein expression

HNE-1 cells (1.5 × 10^5^) were seeded into the 6-well plates and incubated at 37 °C in 5% CO_2_ for 12 h. After that, various formulations (PBS, blank Tf-HPAA-GO, Tf-HPAA-GO/pMMP-9 complex with a weight ratio of 20:1 and PEI-25k/pMMP-9 complex with a weight ratio of 1.3:1) were added, respectively, and then incubated with the HNE-1 cells for 36 h. Subsequently, the cells were washed with PBS twice and the precipitate was added by 100 µL lysis buffer (constitute by 150 mM NaCl, 1.5 mM MgCl_2_, 1 mM EDTA, 50 mM Tris-HCl, pH of 7.4, 1% Triton X-100 and 10% glycerol). The cell lysates were then incubated on ice for 30 min and vortexed every 5 min. The lysates were clarified by centrifugation for at 12,000 rpm 10 min, and the supernatant was boiled for 10 min in loading buffer. On a 12% PAGE-SDS gel, the total protein (20 µL) was separated and then transferred to the PVDF membrane (Bio-Rad). After incubated in 5% BSA in PBS containing Tween-20 for 1 h, the membrane was then incubated in 5% BSA of PBST with MMP-9 antibodies (1:1000) overnight at 4 °C. After the incubation in 5% BSA of PBST with goat anti-rabbit IgG-HRP antibody (1:5000) for 60 min at 25 °C, band was visualized using the ECL system (Pierce). Relative protein expression values were determined using Image-J software.

### 2.8. Co-delivery of DOC and pMMP-9

HNE-1 cells were cultured into a 96-well plate (8 × 10^3^ cells/well) in complete DMEM at 37 °C in a humidified atmosphere with5% CO_2_. After 12 h incubation, the medium was replaced by 100 µL of complete DMEM containing the desired amount of formulations such as the blank Tf-HPAA-GO, Tf-HPAA-GO/DOC (DOC: 1 µg/well), Tf-HPAA-GO/pMMP-9 (weight ratio of 20; pMMP-9: 0.65 µg/well) and Tf-HPAA-GO/DOC/pMMP-9 (weight ratio of 15; DOC of 1 µg/well; pMMP-9 of 0.65 µg/well), and every formulation was set for five multiple holes. Cells treated with the same amount of PBS and HPAA-GO/DOC/pMMP-9 complexes were used as the control groups. After 36 h incubation, the cells were incubated in 100 μL of DMEM containing CCK-8 for another 2 h. The absorbance in each well was measured at a wavelength of 450 nm to calculate the number of viable cells.

### 2.9. *In vivo* assays

For *in vivo* antitumor tests, the HNE-1 tumor-bearing nude mice were modeled and then randomly divided into seven groups (*n* = 5). Subsequently, the mice were injected intravenously using 200 μL Tf-HPAA-GO/DOC/pMMP-9 (pMMP-9 dose of 3.25 μg/g and DOC dose of 5 μg/g). PBS (200 μL), Tf-HPAA-GO/DOC (5 μg/g DOC in 200 μL PBS), free DOC (5 μg/g in 200 μL PBS), Tf-HPAA-GO/pMMP-9 (pMMP-9 dose of 3.25 μg/g in 200 μL PBS), HPAA-GO/DOC/pMMP-9 (pMMP-9 dose of 3.25 μg/g and DOC dose of 5 μg/g), and blank Tf-HPAA-GO (200 μL) were set as the control groups. Particularly, all formulations were injected every two days. After 3 weeks, the animals were sacrificed. The tumor volume was calculated as: *V* = (*L* × *W* ^2^)/2, where *W* and *L* denote the shortest and longest diameters of the tumors, respectively.

### 2.10. Blood compatibility

#### 2.10.1. Hemolysis

For hemolysis testing, the fresh whole blood was taken from mice using the sodium citrate as an anticoagulant with a blood/anticoagulant ratio of 9:1. The whole blood was immediately centrifuged at 1000 rpm for 5 min to remove the resultant buffy coat layer and plasma. The obtained red blood cells (RBCs) were washed with PBS (pH = 7.4) for three times. After that, the RBCs were resuspended in PBS at 16% hematocrit (v/v) and then mixed with 5 mL PBS containing Tf-HPAA-GO at different concentrations (1, 10, 100, and 1000 μg/mL, respectively) for 12 h. The positive (100% hemolysis induced by PBS containing 5 mL 0.1% Na_2_CO_3_ solution) and negative (0% hemolysis of only PBS) controls were set up. Each sample was measured for three times. After the incubation, the RBC suspensions were centrifuged at 1000 rpm for 5 min, and the supernatants were measured for the absorbance at 540 nm (Zhen et al., 2015). The percentage hemolysis was calculated by the optical density (OD) values as the following formula:

Hemolysis(%)=[(OD of the test sample – OD of negative control)×100]/OD of positive control.

#### 2.10.2. Activated partial thromboplastin time (APTT) and prothrombin time (PT) assays

The APTT and PT assays were recorded by the SF-8000 automatic coagulation analyzer (Beijing Succeeder Company, China) with the corresponding reagents provided by the Southern Medical University (Guangzhou, China). Platelet-poor plasma was obtained by centrifuging the citrated whole blood at 1000 rpm for 15 min and then mixed with Tf-HPAA-GO at different concentrations (1, 10, 100, and 1000 μg/mL, respectively). Then, APTT and PT were analyzed at 37 °C. Each experiment was repeated for three times. The sample of platelet-poor plasma mixing with only PBS was set as control.

#### 2.10.3. Cytotoxicity to 3T3 cells

The cytotoxicity of Tf-HPAA-GO to 3T3 cells was determined by CCK-8 assay *in vitro*. The 3T3 cells were cultured onto a 96-well plate (7 × 103 cells per well) in complete DMEM (with 10% fetal bovine serum supplemented and high glucose) in a humidified atmosphere of 5% CO_2_ at 37 °C for 12 h. The growth medium was then replaced with 200 µL DMEM containing the desired amount of Tf-HAA-GO, and five multiple holes were set for every sample. The PBS was used as the control group. After 24 h cultivation, 100 µL of DMEM with 10 µL of CCK-8 were added and then incubated for another 2 h. The cell viability was determined at a wavelength of 450 nm by a microplate reader.

#### 2.10.4. *In vivo* toxicity

For *in vivo* toxicity study, the above nude mice after 21 days *in vivo* assays were sacrificed, and their major organs (heart, liver, spleen, lung, and kidney) were harvested and washed with PBS. After fixed by 4% formaldehyde, histological examination was carried out.

### 2.11. Statistical analysis

The comparison among different groups was analyzed by the one-tailed Student’s *t*-test using the statistical software SPSS 11.5. All data are presented as means ± SD. The difference with *p* < .05 (*) was considered statistically significant.

## 3. Results and discussion

### 3.1. Tf-HPAA-GO preparation

**Scheme 1.**
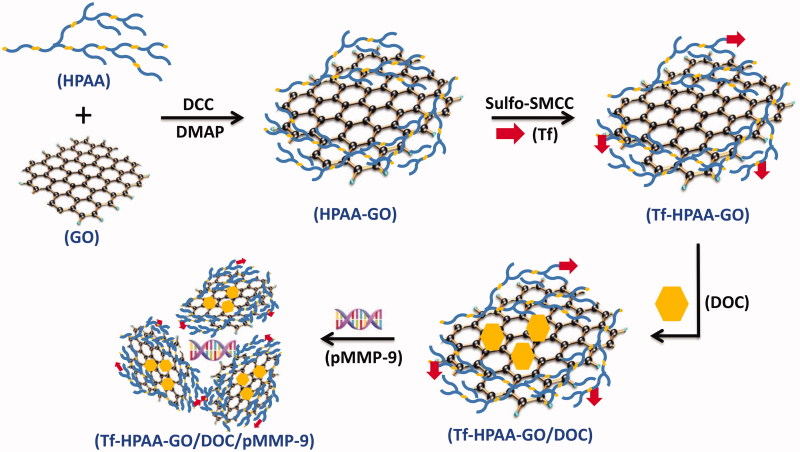
The preparation of Tf-HPAA-GO/DOC/pMMP-9.

The preparation routes to Tf-HPAA-GO were shown as Scheme 1. To obtain the self-sensible drug and gene co-delivery system, the GSH-response HPAA was firstly synthesized according to the reported work (Li et al., 2016), and then GO was conjugated with HPAA through the aminification reaction. Then, the target protein molecule of Tf containing mercapto group was conjugated with the amino group of HPAA to form the Tf-HPAA-GO carrier using sulfo-SMCC as the activity. After that, the Tf-HPAA-GO carrier could co-deliver GO and pMMP-9 through the π-π interactions between GO and DOC and the electrostatic interactions between HPAA and pMMP-9.

**Figure 1.**
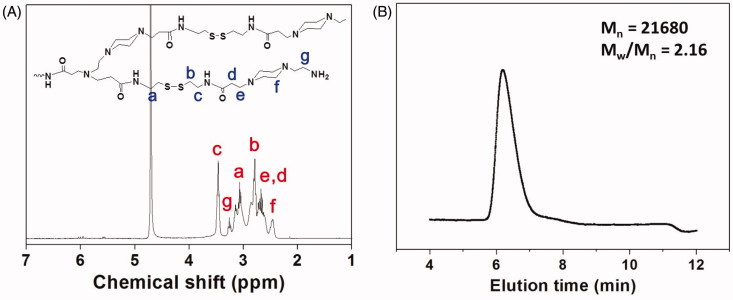
(A) ^1^H NMR spectrum of HPAA (25 °C, D_2_O). (B) GPC trace of HPAA (37 °C, aqueous 0.8 mol/L NaNO_3_).

The chemical structure of the synthesized HPAA was characterized by ^1^H NMR. As shown Figure 1(A), all characterized peaks have been marked corresponding with its chemical structure, indicating that HPAA was synthesized successfully. The molecular weight and molecular weight distribution of HPAA have also been determined by GPC analysis. As shown in Figure 1(B), HPAA displayed the molecular weight of *M_n_* = 21,680 and *M_w_*/*M_n_* = 2.16, using poly(ethylene oxide) as the standard.

**Figure 2.**
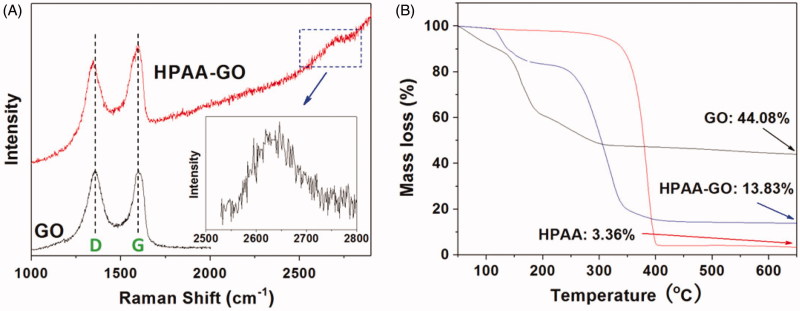
(A) Raman spectra of GO and HPAA-GO. (B) TG curves of GO, HPAA, and HPAA-GO.

After conjugated with GO, the obtained HPAA-GO was characterized by Raman spectra to confirm its chemical structure. As shown in Figure 2(A), both GO and HPAA-GO showed their characterized peaks at about 1595 cm^−1^ and 1349 cm^−1^, which were the typical G and D bands for the carbon materials. After HPAA conjugation, the D band of HPAA-GO showed a blue shift and the intensity ratio of G/D increased compared with that of GO (G/D increased from 1.02 to 1.05 after conjugated with HPAA). This result implied that GO was chemical modified by HPAA and then resulted in the larger defects in origin GO structure (Gu et al., 2017). Moreover, a small peak named G’ or two D band was found at about 2600–2700 cm^−1^, which could be used to calculate the number of GO layers for HPAA-GO. From the result shown in Figure 2(A) insert, it was determined that GO showed a monolayer structure in HPAA-GO (Ferrari et al., 2006). The composition of HPAA-GO could be calculated by TG analysis of the residual mass and the results were shown in Figure 2(B). It was found that GO remained about 44.08% mass residues after heated to 650 °C under the nitrogen atmosphere, while HPAA only remained 3.36% residues. For HPAA-GO, it remained about 13.83% residues in the end. From these results, the content of HPAA in HPAA-GO could be determined to be about 74.3 wt%.

To improve the drug and gene co-delivery ability, Tf, a targeting molecular to HNE-1 cells (Liu et al., 2018), was conjugated with HPAA-GO to form the Tf-HPAA-GO, and the Tf content in Tf-HPAA-GO was determined as 2.3 wt% after analyzed by the Micro BCA Protein Assay Kit.

**Figure 3.**
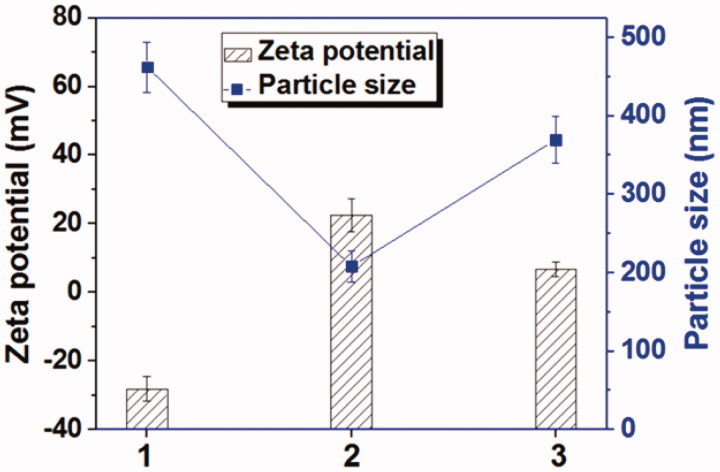
The zeta potentials and particle sizes of GO, Tf-HPAA-GO, and Tf-HPAA-GO in the presence of 10 mmol/L GSH (37 °C for 4 h) (1: GO; 2: Tf-HPAA-GO; 3: Tf-HPAA-GO mixed with 10 mmol/L GSH).

HPAA segments could be degraded in the availability of GSH which was rich in most tumor cells. This peculiarity makes it an excellent drug delivery system, because HPAA could be degraded intracellular and therefore the loaded drugs could be released sufficiently (Gu et al., 2017). The GSH-response of Tf-HPAA-GO was characterized by zeta potentials and particle sizes with or without GSH incubation. As shown in Figure 3, GO showed a zeta potential of –28.2 mV due to abundant carboxyl groups itself, and its particle size was larger than 450 nm due to the aggregation of GO sheet. After conjugated with HPAA, the Tf-HPAA-GO showed a good dispersion in aqueous solution with the particle size of 225 nm, and the zeta potential was +24.6 mV due to the cationic HPAA. After incubating with 10 mmol/L GSH for 4 h, the zeta potential of Tf-HPAA-GO reduced to +8.6 mV while the particle size increased to 370 nm, indicating that the dispersion of Tf-HPAA-GO was destroyed in a certain extent. These results indicated that Tf-HPAA-GO could be degraded in aqueous GSH solution and may deliver drugs efficiently into tumor cells.

### 3.2. DOC delivery

As the clinical drug for NPC therapy, DOC was loaded on Tf-HPAA-GO to form the Tf-HPAA-GO/DOC complex through the π-π interactions between GO and DOC. It has been reported that the drugs containing benzene groups could stack on the GO plane effectively, so GO showed higher drugs loading amount than micelles and other usual carriers (Ma et al., 2012; Rahmani et al., 2018; Liu et al., 2018). In this work, the loading amount of DOC in Tf-HPAA-GO/DOC complex was determined as 5.6% (w/w) by HPLC analysis, much higher than our previous work (Liu et al., 2016).

**Figure 4.**
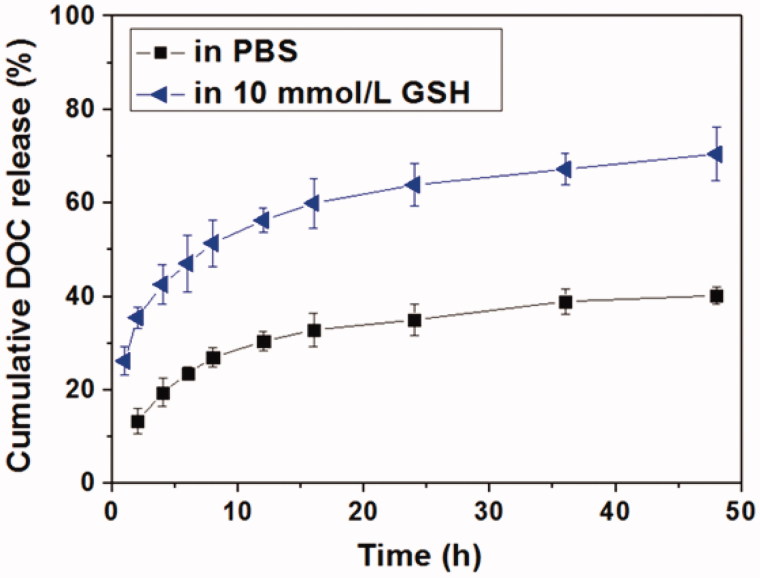
*In vitro* DOC release profiles from Tf-HPAA-GO/DOC complex in PBS or aqueous 10 mmol/L GSH solution (37 °C).

The *in vitro* DOC release behaviors in the absence or presence of GSH were shown in Figure 4. It could be seen that DOC could be released slowly in PBS and about 40% of the loaded DOC was released after 48 h. Correspondingly, DOC was released much faster in the presence of GSH, indicating a GSH-responsive manner. The cumulative DOC release amount within 48 h in the presence of 10 mM GSH was up to 67.8%, suggesting the DOC release was accelerated by the degradation of HPAA. The degradation of HPAA segments made the Tf-HPAA-GO/DOC complex lost the barrier of the periphery and then speed up the DOC molecule diffusion. Moreover, it has been revealed that GSH overexpresses in most tumor organs and cells, and in cytoplasm the GSH concentration can reach 10 mM (Pan et al., 2012), which is sufficient for the degradation of HPAA segments of the Tf-HPAA-GO/DOC complex in this work. This result indicated that such a Tf-HPAA-GO carrier may enable the high therapeutic level in tumor locations with considerably reduced toxicity to normal tissues and displaying an “on-demand” drug release manner.

**Figure 5.**
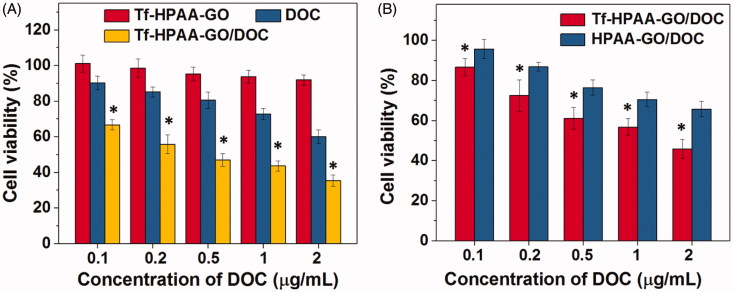
(A) HNE-1 cells viability after incubated with blank Tf-HPAA-GO, free DOC and Tf-HPAA-GO/DOC for 24 h (37 °C and 5% CO_2_, *n* = 5). (B) HNE-1 cells viability after incubated with Tf-HPAA-GO/DOC and HPAA-GO/DOC for 4 h (37 °C and 5% CO_2_, *n* = 5).

The *in vitro* tumor cells inhibition assays were performed on HNE-1 cells. Figure 5(A) showed the results of HNE-1 cells viability after incubated with blank Tf-HPAA-GO, free DOC, and Tf-HPAA-GO/DOC for 24 h. It was found that blank Tf-HPAA-GO showed noncytotoxicity to HNE-1 cells in the experimental concentrations, indicating a good biocompatibility of Tf-HPAA-GO. Free DOC and Tf-HPAA-GO/DOC showed the obvious cytotoxicity to HNE-1 cells, and the cell viability decreased with the increase of concentrations, displaying the concentration-dependent inhibition effect. In particularly, Tf-HPAA-GO/DOC showed much better inhibiting effect than that of free DOC on HNE-1 cells. For example, the Tf-HPAA-GO/DOC complex showed the inhibition efficiency of 52.8% to HNE-1 cells at the DOC concentration of 0.5 µg/mL, at which the free DOC showed only 20.6% inhibition efficiency. According to the inhibition results, the IC_50_ of the free DOC and Tf-HPAA-GO/DOC to HNE-1 cells were determined as 3.82 µg/mL and 0.55 µg/mL (concentration of DOC), respectively, implying that Tf-HPAA-GO/DOC displayed much better therapeutic effect than free DOC to HNE-1 tumor, which may be relative to the self-sensibilization effect of Tf-HPAA-GO/DOC. The excellent effect of Tf-HPAA-GO/DOC on HNE-1 tumor cells may be attributed from its GSH-response ability of HPAA segments, which resulted in consuming the intracellular GSH and then improving the sensibility of tumor cells to the chemotherapy drugs. Tang et al. have reported that the HPAA segments could be degraded intracellularly and consume the intracellular GSH, and then the GSH expression could be down-regulated (Tang et al., 2018). The down-regulated GSH could improve the sensibility of some tumor cells to chemotherapy drugs, and this strategy has been used in clinical tumor therapy. So, in this work, the drug carrier containing HPAA segments could improve the sensitivity of HNE-1 cells to DOC and showed the self-sensibilization effect of the drug carrier.

To further verify the targeting ability of Tf to HNE-1 cells, the cells were incubated with Tf-HPAA-GO/DOC and HPAA-GO/DOC for 4 h and then replaced by DMEM for another 24 h. The cell viability results were shown as Figure 5(B). It was found that both Tf-HPAA-GO/DOC and HPAA-GO/DOC showed the concentration-dependent cytotoxicity to HNE-1 cells and the Tf-HPAA-GO/DOC showed significantly better inhibition effect than HPAA-GO/DOC. This result confirmed that Tf was targeting to HNE-1 cells and Tf-HPAA-GO/DOC could be uptaken by HNE-1 cells easier than HPAA-GO/DOC.

### 3.3. pMMP-9 delivery

**Figure 6.**
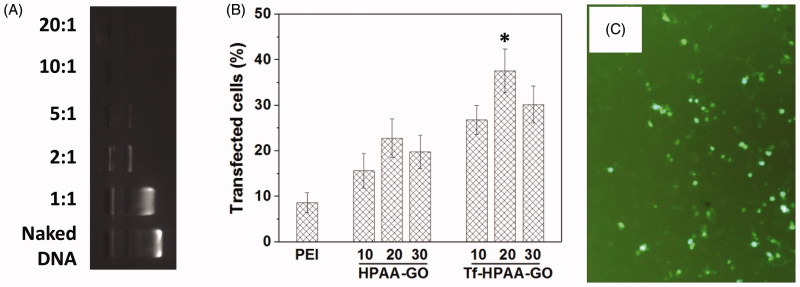
(A) Gel electrophoresis assay of Tf-HPAA-GO/pMMP-9 complexes with different weight ratios. (B) Gene transfection results of HNE-1 cells treated with PEI-25k/pMMP-9 (w/w = 1.3:1), HPAA-GO/pMMP-9, and Tf-HPAA-GO/pMMP-9 (w/w = 10, 20, and 30, respectively) complexes (*n* = 3). (C) Typical image of transfected HNE-1 cells with Tf-HPAA-GO/pMMP-9 at a weight ratio of 20.

HPAA has been developed as an efficient gene delivery vector benefiting from its hyperbranched structures and intracellular biodegradation (Li et al., 2016). To confirm the pMMP-9 delivery ability of Tf-HPAA-GO, gel retardation assay was firstly performed to examine its gene condensation ability. As shown in Figure 6(A), at the low Tf-HPAA-GO/pMMP-9 weight ratio of 1:1 to 5:1, pMMP-9 could be observed to move out under the experimental electric field conditions. Above the ratio of 10:1, pMMP-9 was totally retarded, implying that the pMMP-9 was completely condensed by Tf-HPAA-GO, suggesting Tf-HPAA-GO showed the good gene condensation ability *in vitro*.

The gene transfection efficiency of Tf-HPAA-GO/pMMP-9 complexes at different weight ratios was evaluated by *in vitro* transfection assay, and PEI-25k/pMMP-9 (w/w = 1.3) and HPAA-GO/pMMP-9 were used as the positive controls. As shown in Figure 6(B,C), both Tf-HPAA-GO and HPAA-GO showed the considerable gene delivery ability to MMP-9. Particularly, both of them showed the significant higher transfection efficiency than PEI-25k/MMP-9 in the presence of serum. Their high transfection efficiency may arise from two reasons: one is that HPAA displayed better blood compatibility than PEI due to its chemical structure, which made it the relative low zeta potential and then made its complexes more stable in the serum; the other is that its hyperbranched structure and bioreducible disulfide linkages could bind gene easily and form the stable complexes extracellularly, while degraded intracellularly and released the gene rapidly to complete the transfection. Moreover, Tf-HPAA-GO showed the significant higher transfection efficiency than HPAA-GO, confirming the good targeting ability of Tf to HNE-1 cells. Then, the Tf-HPAA-GO was designed successfully for delivering gene to HNE-1 cells. At the weight ratio of 20, Tf-HPAA-GO showed the best transfection ability in this work, and about 37% HNE-1 cells could be transfected.

**Figure 7.**
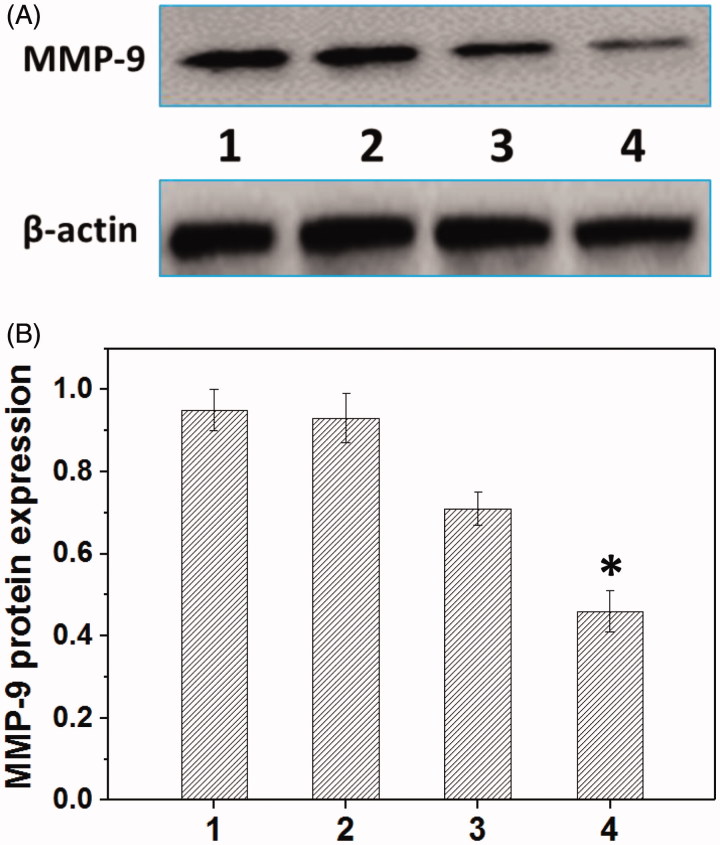
Representative MMP-9 protein expression determined by western blot analysis (A) and its quantitative analysis of MMP-9 protein expression (B) in HNE-1 cells treated with different formulations (*n* = 3). (1: PBS control; 2: blank Tf-HPAA-GO; 3: PEI-25k/pMMP-9 at a weight ratio of 1.3; 4: Tf-HPAA-GO/pMMP-9 at a weight ratio of 20).

Metastasis-related recurrence responsible for the majority of the mortality is a common occurrence in HNE-1 cancer, which is similar to most reported tumor diseases (Oh et al., 2017), so the inhibition of migration-related signals, such as MMP-9 protein, has been an effective strategy for cancer treatments. The MMP-9 protein expression in HNE-1 cells treated with various formulations was analyzed by the western blot assay (Jiang et al., 2017). As shown in Figure 7(A,B), the blank Tf-HPAA-GO showed the negligible effect on MMP-9 protein expression in HNE-1 cells, while the formulations loading pMMP-9 (PEI-25k/pMMP-9 and Tf-HPAA-GO/pMMP-9) showed the significant down-regulation of MMP-9 protein expression. Particularly, in accordance with the transfection assay results, Tf-HPAA-GO/pMMP-9 showed the best result on the suppression of MMP-9 protein expression, further confirming the good gene delivery ability of Tf-HPAA-GO.

### 3.4. Co-delivery

**Figure 8.**
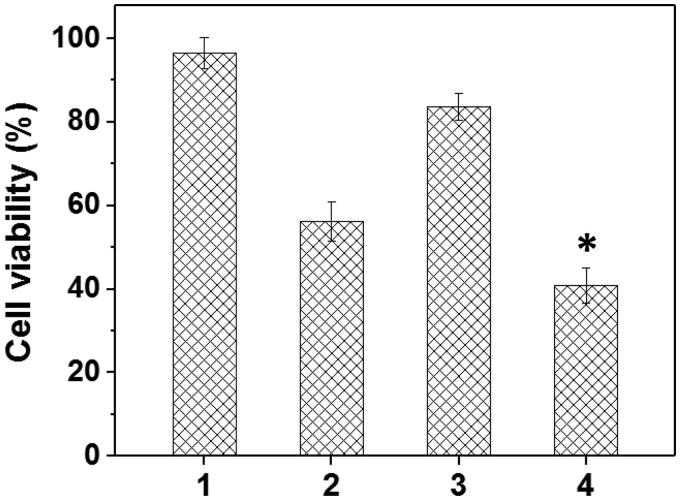
The HNE-1 cell viability treated with different formulations (*n* = 5) (1: blank Tf-HPAA-GO; 2: Tf-HPAA-GO/DOC (1 µg/well DOC); 3: Tf-HPAA-GO/pMMP-9 (weight ratio of 20, 0.65 µg/well pMMP-9); 4: Tf-HPAA-GO/DOC/pMMP-9 (weight ratio of 20, 1 µg/well DOC, 0.65 µg/well pMMP-9)).

To verify the drug/gene co-delivery strategy for HNE-1 tumor therapy, *in vitro* CCK-8 assay was firstly carried out to verify the combined inhibition effect of pMMP-9 and DOC. As shown in Figure 8, the cells treated with blank Tf-HPAA-GO showed the negligible inhibition effect on HNE-1 cells, indicating the good biocompatibility of the co-delivery system. The formulations containing pMMP-9 or DOC showed the significant cytotoxicity to HNE-1 cells, and Tf-HPAA-GO/DOC displayed 52% inhibition ratio and Tf-HPAA-GO/pMMP-9 displayed 15% inhibition ratio respectively. For the co-delivery of Tf-HPAA-GO/DOC/pMMP-9, it showed the best inhibition effect and more than 64% HNE-1 cells were inhibited, significantly higher than that of pMMP-9 or DOC used only. This result indicated that the drug/gene co-delivery strategy displayed the outstanding inhibition effect on HNE-1 cells and had the potential application in combined tumor therapy.

**Figure 9.**
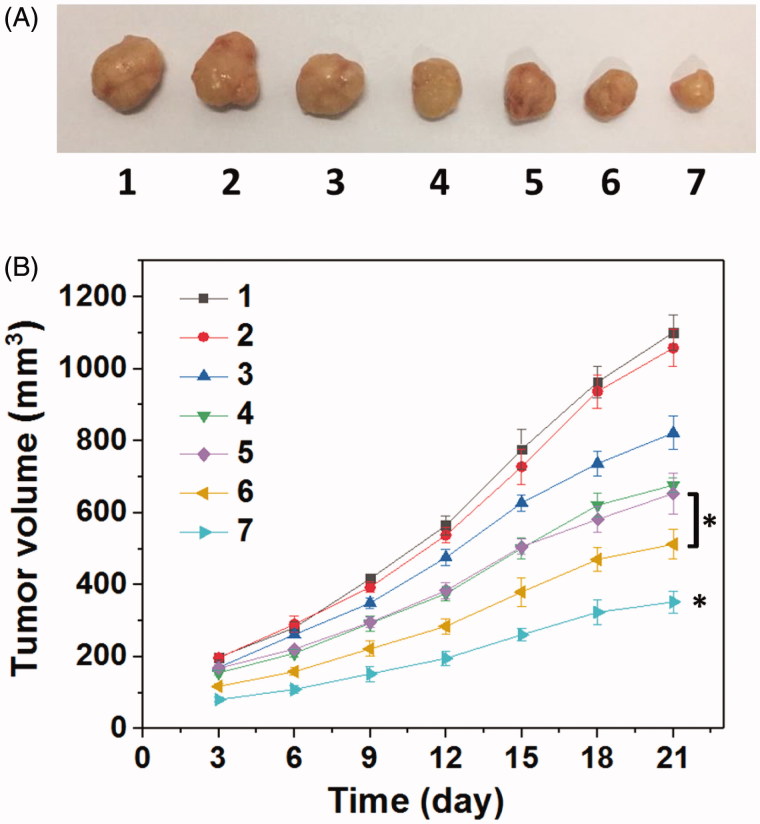
(A) Representative image of HNE-1 tumors at the 21st day and (B) tumor growth profiles treated with different formulations (*n* = 5). (1: PBS control; 2: blank Tf-HPAA-GO; 3: Tf-HPAA-GO/pMMP-9; 4: HPAA-GO/DOC/pMMP-9; 5: free DOC (docetaxel injection, purchased from Rhone-Poulenc Rorer S.A.); 6: Tf-HPAA-GO/DOC; 7: Tf-HPAA-GO/DOC/pMMP-9).

Encouraged by the well-combined therapeupic effect *in vitro*, the *in vivo* antitumor assay was then investigated through the treatment of the nude mice bearing HNE-1 tumor. Figure 9 gave the tumors growth profiles and the representative tumors image treated with various formulations. It was found that the blank Tf-HPAA-GO showed no effect on HNE-1 tumor growth and there was no significant difference in growth profiles with PBS control. The other groups treated with DOC or pMMP-9 formulations displayed the obvious antitumor effect. Particularly, the tumors treated with Tf-HPAA-GO/DOC/pMMP-9 showed the smallest volume and slowest growth profile, which was a significant difference with other groups. This result indicated that the drug and gene co-delivery system showed the potential application in combined tumors therapy. Moreover, there was a significant difference between Tf-HPAA-GO/DOC/pMMP-9 and HPAA-GO/DOC/pMMP-9, implying the good targeting ability of Tf *in vivo*. In addition, compared with free DOC injection, Tf-HPAA-GO/DOC also showed much better antitumor effect. This result was benefited from the self-sensibilization effect of Tf-HPAA-GO to a certain degree. These results further confirmed that the self-sensibilization Tf-HPAA-GO carrier combining the drug and gene co-delivery strategy may be a promising treatment to cancers.

### 3.5. Biocompatibility

**Figure 10.**
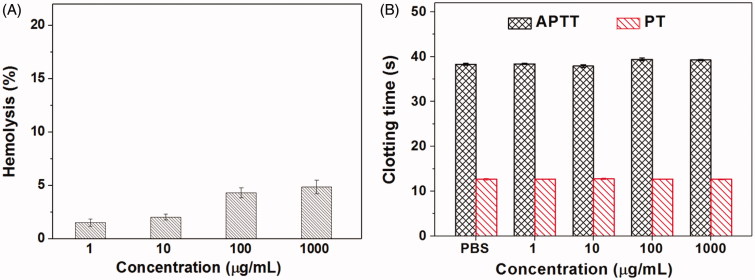
The hemolysis (A) and clotting analysis (B) of Tf-HPAA-GO (*n* = 5).

For drug delivery systems, their blood compatibility is a concern. Their interactions with the compositions of blood are considered as the serious limitation in clinical, and their nonspecific interactions could even severely diminish the half-life and targeting of drugs (Kumar et al., 2017). The blood compatibility of Tf-HPAA-GO was assessed by spectrophotometric measurement of hemoglobin released from erythrocytes after Tf-HPAA-GO treatment. Figure 10(A) showed the percentage hemolysis of the blood in contact with different Tf-HPAA-GO concentrations. It was found that CS-PLLD-Tf exhibited the good blood compatibility. Even the concentration reached to 1 mg/mL, the sample showed a nonhemolytic effect with the extent of hemolysis lower than the permissible level of 5% (Davoudi et al., 2017).

Another important concern on blood compatibility for drug carriers is the effect on the blood coagulation (Chai et al., 2017). Coagulation at the right time and location is necessary to maintain normal metabolism, while inappropriate coagulation will cause severe, even fatal, risks to the living system. Therefore, the effect of Tf-HPAA-GO on coagulation is a key factor in the blood safety evaluation. The blood coagulation cascade contains three types of pathways: intrinsic, extrinsic, and common pathway. Thereinto, the performance of the intrinsic and common coagulation pathways are measured by APTT, which refers to the time needed for forming a fibrin clot after a partial thromboplastin reagent or CaCl_2_ is added. Meanwhile, PT measures the performance of both extrinsic and common coagulation pathways, and refers to the time taken to form a fibrin clot after tissue thromboplastin is added. The effects of the Tf-HPAA-GO on APTT and PT are shown in Figure 10(B). Compared with the PBS control, Tf-HPAA-GO did not significantly change the APTT and PT of the blood under the concentration of 1 mg/mL. The results indicated that Tf-HPAA-GO under experimental concentrations had no obvious activation to coagulation factor XII in the plasma and thrombin, suggesting the blood safety in this work. The good blood compatibility of Tf-HPAA-GO may be attributed from its hyperbranched structure and low cationic charge density, which resulted in it showed the relative low zeta potential than other cationic polymer such as PEI, and then was avoided to interact with the proteins in the blood (Ma et al., 2014).

**Figure 11.**
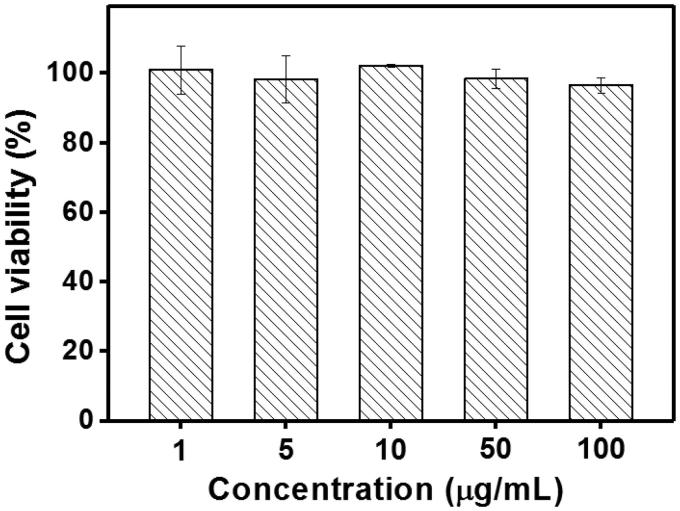
Cytotoxicity of Tf-HPAA-GO to 3T3 cells (37 °C and 5% CO_2_, *n* = 5).

The cytotoxicity of the blank Tf-HPAA-GO was also evaluated using 3T3 cells to confirm its safety, and the result was shown in Figure 11. It was found that the cell viability of the 3T3 cells treated with different concentration of Tf-HPAA-GO for 24 h remained constant and there was no significant difference with PBS control. Even the concentration of Tf-HPAA-GO reached to 100 µg/mL, the 3T3 was still viable and no significant difference was found compared with PBS control. This result indicated that Tf-HPAA-GO showed good safety.

**Figure 12.**
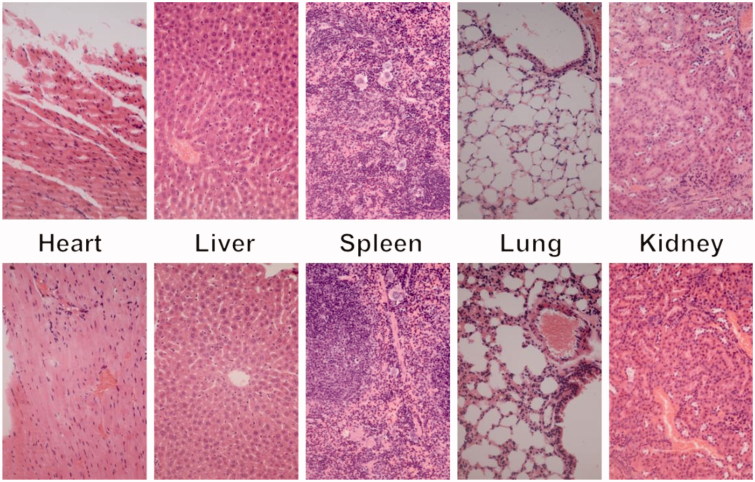
Representative HE stain image of organ histology by Tf-HPAA-GO/DOC/pMMP-9 (top row) and PBS control (bottom row).

*In vivo* toxicity assay was also performed through a histological analysis to prove the safety of Tf-HPAA-GO. As shown in Figure 12, histologically, no visible difference was observed between the two groups. The *in vivo* toxicity of polymers is influenced by the chemical structures, size, biodistribution, and metabolism as well as the surface and terminal groups (Li et al., 2017). The nonobserved toxicity of Tf-HPAA-GO could be attributed to its hyperbranched molecular structure and the degradation ability, which could reduce the cytotoxicity of polymers compared with a linear structure having the similar molecular weights.

## 4. Conclusion

To combine the sensitized chemotherapy with gene therapy for NPC treatment, the GSH-response cationic HPAA was used to modify GO, and Tf was also conjugated to target NPC cells. The carrier of Tf-HPAA-GO displayed the GSH-response and could accelerate DOC release in aqueous GSH solution. More importantly, it displayed much better inhibition effect to HNE-1 cells than free DOC, which was confirmed that the chemotherapeutic self-sensibilization effect. Meanwhile, Tf-HPAA-GO simultaneously possessed excellent pMMP-9 delivery with significant down-regulation of MMP-9 protein expression. Benefiting from the self-sensibilization effect as well as the high-efficiency drug and gene delivery, Tf-HPAA-GO/DOC/pMMP-9 showed significantly improved therapeutic efficacy to NPC *in vitro* and *in vivo*. The work provided a facile platform to integrate the drug/gene co-delivery strategy, self-sensibilization and targeting effect into one single nanocomposite for potential cancer treatment.
